# Polymorphism and Microstructural Changes in Avocado Pulp (*Persea americana* Mill.) After Scraped-Surface Heat Exchanger Processing

**DOI:** 10.3390/foods13233717

**Published:** 2024-11-21

**Authors:** Amanda Valle-Gómez, Raúl Borja-Urby, Alicia Ortiz-Moreno, Darío Iker Téllez-Medina

**Affiliations:** 1 Departamento de Ingeniería Bioquímica, Escuela Nacional de Ciencias Biológicas, Instituto Politécnico Nacional, Unidad Profesional Adolfo López Mateos, Zacatenco, Av. Wilfrido Massieu 399, Col. Nueva Industrial Vallejo, Gustavo A. Madero, Ciudad de México 07738, Mexico; avalleg1600@alumno.ipn.mx (A.V.-G.); aortizm@ipn.mx (A.O.-M.); 2 Centro de Nanociencias y Micro y Nanotecnologías, Instituto Politécnico Nacional, Wilfrido Massieu s/n, UPALM, Gustavo A. Madero, Ciudad de Mexico 07738, Mexico; rborjau@ipn.mx

**Keywords:** polymorphism, X-ray diffraction, HRTEM, avocado, scraped-surface heat exchanger

## Abstract

Avocado (*Persea americana* Mill.) is a fruit with a high content of unsaturated fatty acids and bioactive compounds, whose consumption has considerably increased in the USA and Europe. Thus, the conservation of the avocado mesocarp (pulp) has become more relevant. Avocado pulp was processed using a scraped-surface heat exchanger (SSHE) system to extend the shelf-life of the mesocarp. Through analysis with X-ray diffraction and HRTEM, it was possible to identify crystalline-type structures in the avocado pulp processed and stored at 4 °C. The 2θ-angles and d-spacing of the structures that reported the highest diffraction intensity are comparable to the polymorphs β′ reported in the literature for fatty acid mixtures processed under similar conditions. Furthermore, the X-ray signals suggest the presence of polymorphs α and β in all samples processed and stored at different temperatures. Calorimetry analysis showed curves with first-order phase changes as indicative of crystallization-type transitions. The shelf-life evaluation of the avocado pulp showed that the crystallization process minimized the losses of antioxidant capacity and prevented color change, while the enzyme polyphenol oxidase remained inactivated. The changes induced by the SSHE continuous processing applied might represent an alternative to obtaining avocado products that preserve avocado’s properties and extend its shelf-life.

## 1. Introduction

The main world producer of avocado is Mexico [1,2], and the most consumed variety of this fruit is Hass [3]. Avocado is a fruit that prioritizes, during maturation, the accumulation of oil in its idioblastic cells [3,4]. The highest concentration of fatty acids present in avocado relates to monounsaturated fatty acids [5,6], which are associated with the prevention of diabetes and cardiovascular diseases and with the increase in high-density lipoprotein levels in blood which are associated with being a protective against coronary heart disease [7,8,9]. The highest fatty acids in avocado oil are oleic and linoleic, though the content ratio between oleic and linoleic acid is in the order of 8:1 [1,10,11]. The oil content reaches 60–70% of the dry weight of the avocado pulp, while the content of carbohydrates is only 10% [3,12].

The consumption of avocado pulp has increased in recent years, not only because of the presence of unsaturated acids but also due to its content of bioactive compounds and the impact of the latter on human health. Pigments such as chlorophylls and carotenoids, whose biological activities are associated with the prevention of cancer and anemic processes, as well as with cell protective mechanisms [12,13], are present in significant amounts in the avocado pulp. Furthermore, the fruit has phenolic compounds such as vanillic, *p*-coumaric, benzoic, caffeic, ferulic, and chlorogenic acids, epicatechin, catechin, and alpha and beta tocopherols. These phenolic compounds have antimicrobial, cardioprotective, antiallergenic, and anti-inflammatory activities [14,15,16]. The highest concentration of phytosterols present in avocado pulp is associated with β-sitosterol, followed by campesterol and stigmasterol; these compounds contribute to the inhibition of the generation of carcinogenic compounds and to the decrease in the activity of cholesterol [17,18,19,20].

Research on the thermal and non-thermal process of avocado pulp tends to overlook the loss of the bioactive compounds and to concentrate on extending the shelf-life of the mesocarp. Nevertheless, the thermal inactivation of the polyphenol oxidase enzyme is another aspect to consider for the final product since this enzyme causes oxidation and color changes in the pulp [21].

The high-pressure process (HHP) is the most recurrent method for preserving avocado pulp [22]. An HHP is a non-thermal technology and has no significant impact on the color, flavor, and nutritional quality of the food matrix treated [23]. This process decreases the activity of enzymes and microbial load. However, during storage, the avocado puree develops color changes [24]. Freeze-drying is another technology employed to extend the shelf-life of avocado pulp. It has been reported to minimize the losses of nutritional value because of the low temperatures of operating conditions; however, freeze-dried pulp shows a higher browning index than fresh avocado pulp [25]. This may be because the polyphenol oxidase needs high temperatures (60–80 °C) to be inactivated [26].

Changing the microstructure of the avocado pulp as a method to extend shelf-life could decrease the loss of bioactive compounds while inactivating enzymes, thus preventing color changes. In products such as ice cream, margarine, or cocoa butter, crystallization improves the functionality of vegetable oils and gives the products smooth and creamy properties. The crystallization of fatty acids derives from polymorphism and molecular interactions [27]. The melting points and crystal packing of fatty acids show the different types of polymorphs. There are three main types of polymorphs: α, β′, and β [27,28,29,30]. The interplanar distances for each type of polymorph have been reported and are useful in identifying these structures; Table 1 summarizes the ranges of interplanar distances among these polymorphic structures. The long spacings are around 2θ = 1–15°, and the short spacings show a range of 2θ = 16–25°. The short spacing quantification gives the identification of the polymorphic structures [27,31].

The operating conditions affect the nucleation rate, crystal growth, and ratio of the type of polymorphs [32]. The different operating conditions can be chosen depending on the composition of the matrix, and their interactions of different compounds. For example, in the case of the rate of cooling, it is desirable to have rapid cooling and uniformity in the food matrix, because the production of nuclei is maximized and the size distribution of the crystal becomes narrower. In the case of lipid products, the crystallization is directly related to high- and low-melting components and affects the size, crystalline mass, and the polymorph formed [32,33]. The temperature is important for the nuclei formation. The balance between the thermodynamic force (supersaturation) and mobility effects (glass transition) is the optimal point of the nucleation phase, correlated with the maximum number of crystals formed [32]. For instance, changes in the melting temperatures and onset of crystallization of tempered and untempered cocoa butter were observed by Ray et al. (2012) [34]. Nosratpour et al., (2020) [35] observed different microstructural behavior under different cooling rates of milkfat blends. Given the avocado pulp has significant contents of fatty acids, the crystallization process could improve the texture and characteristics of the final product.

In the food industry, the scraped-surface heat exchanger (SSHE) works with fluids with a high viscosity and density [31], such as ice cream and related products, in crystallization processing [36]. SSHEs consist of a double-cylinder with an insider rotor and have blades that scrape the inner wall of the cylinder. The rotation of the rotor generates turbulence on the fluid and improves the heat transfer [36,37]. SSHEs are widely used for crystallization because the continuous system impacts directly on the structure and orientation of crystals, polymorphism, and the distribution of the crystals in the food matrix [38,39].

To the authors’ understanding, this is the first work that investigates the crystallization process to extend the shelf-life of avocado pulp and analyzes the effect of this technology on the avocado pulp microstructure. Hence, the present work aimed to change the microstructure of avocado pulp by using a system of three scraped-surface heat exchangers (heating, pre-cooling, and cooling phases) as a continuous processing technique without affecting the nutritional composition and beneficial properties of avocado pulp and extending its shelf-life.

## 2. Materials and Methods

### 2.1. Materials

The avocado (*Persea americana* Mill.) Hass was purchased from producers in the state of Michoacán, Mexico. Avocados were selected according to homogeneity in maturity; specimens with external and internal damage were discarded. The selected avocados were cleaned and sanitized by rinsing with tap water and NaClO 0.1% dissolution (Hycel, Zapopan, Jalisco, Mexico). The pulp was manually separated from seeds and peels, and the yield of pulp per kilogram of avocado was around 60%. The size reduction in the avocado pulp was performed in a cutter mixer (Crypto Peerless K55, Crypto Peerless, Ltd., Halifax, UK) for 2 min with 50 mL of water per kilogram of pulp.

### 2.2. Reagents

The HPLC-grade methanol, hexane, diethyl ether, and acetone solvents used in the extractions were purchased from JT Baker (Avantor Performance Materials, Inc., Xalostoc, Estado de México, Mexico). The Trolox standard, 2,2-diphenyl-1-picrylhydrazyl (DPPH), Folin–Ciocalteu, and catechol reagents were purchased from Sigma-Aldrich (Sigma-Aldrich, Co., Santa Clara, CA, USA).

### 2.3. Crystallization Process

#### Crystallization Process in SSHE

**Figure 1:**
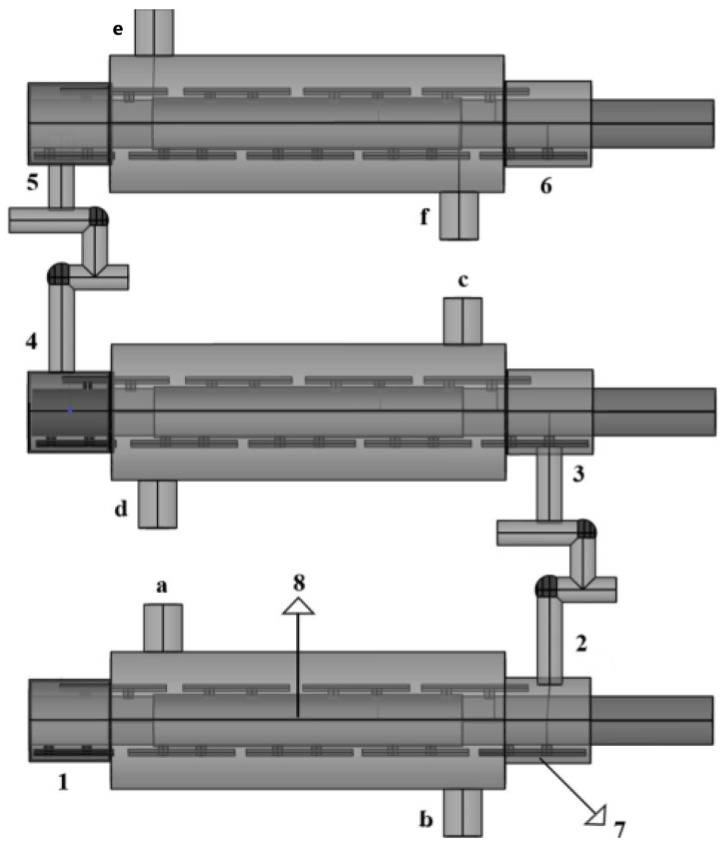
Scraped-surface heat exchanger system, (1) inlet of SSHE heating, (2) outlet of SSHE heating, (3) inlet of SSHE pre-cooling, (4) outlet of SHHE pre-cooling, (5) inlet of SSHE cooling, (6) outlet of SSHE cooling, (7) blades, (8) rotor, (a) outlet of heating medium, (b) inlet of heating medium, (c) inlet of pre-cooling medium, (d) outlet of pre-cooling medium, (e) outlet of cooling medium, (f) inlet of cooling medium.

The process consisted of three coupled scraped-surface heat exchangers for the heating, pre-cooling, and cooling stages. Figure 1 depicts the components of the equipment used. The avocado pulp sample begins the process in the SSHE for heating (1). The blades (7) and the rotation of the rotor (8) enhanced the heat transfer. The operating conditions for the heating, pre-cooling and cooling stages are summarized in Table 2.

The volume flow rate and outlet temperature of avocado pulp were 200 mL/min and 7 °C, respectively, and it was collected and stored under three different temperatures, namely 4 °C, 10 °C, and −20 °C, until analysis (2 weeks approximately). The amount of product obtained per kg of avocado pulp submitted to SSHE processing was 0.4 kg. The storage temperatures were selected by considering common storage temperatures for foodstuff, namely fresh conditioning (10 °C), refrigeration (4 °C), and freezing (−20 °C), in order to prevent microbial growth.

### 2.4. Physicochemical Parameters

#### 2.4.1. Moisture Content

The moisture content was determined by dehydrating 5 g of each sample in triplicate at 110 °C up to constant weight [14,40,41].

#### 2.4.2. Color

The color assessment of avocado pulp at 25 °C and avocado pulp processed and stored at 4 °C was conducted using the CIE coordinates (L*, a*, b*). The read was from a CR-10 tristimulus colorimeter (Konika Minolta, Inc., Tokyo, Japan) with a D75 light source [42]. Color difference (Δ*E*) was calculated from Equation (1):

(1) $$∆E*=\sqrt{{\left(∆L*\right)}^{2}+{\left(∆a*\right)}^{2}+{\left(∆b*\right)}^{2}}$$

#### 2.4.3. Viscosity Measurements

The rheological properties of avocado pulp were determined by using the concentric cylinder configuration of a rheometer (HAAKE RotoVisco 1, Thermo Fisher Scientific, Waltham, MA, USA), specifically the bob Z-31. Samples of 52 g of avocado pulp for each determination were introduced in the corresponding cup. The temperature was maintained with a water bath (HAAKE DC30-K20, Thermo Fisher Scientific, Waltham, MA, USA), at 80 °C, 25 °C, and 5 °C, which were the mean temperatures of each stage of the process inside the SSHE. The behavior of shear stress (*τ*, Pa‧s) with respect to the shear rate $\dot{\gamma }$ (1/s) of a non-Newtonian fluid was used to characterize the consistency (*K*, Pa‧s^n^) and behavior index (*n*, dimensionless) of samples. The viscosity results were obtained by employing the power law model [21,24] (Equation (2)):

(2) $$\tau =K{\left(\dot{\gamma }\right)}^{n}$$

### 2.5. Biochemical Analysis

#### 2.5.1. Avocado Oil Extraction

The Soxhlet method (AOAC 963.15) was employed to determine oil content. Samples of 20 g of avocado pulp were dehydrated at 110 °C up to constant weight. The dried sample was spread in the thimble and placed into the Soxhlet device. The reflux took place with 150 mL of ethyl ether for approximately four hours. The flask was dried at 110 °C to constant weight.

#### 2.5.2. DPPH

The methods reported by Figueroa et al. [43] and Diez Rodilla et al. [44] were used with some modifications. Briefly, samples of 1 g of avocado pulp and 10 mL of an 80:20 (*v*/*v*) aqueous methanolic solution were homogenized in triplicate using a magnetic stirrer plate (Thermo Fisher Scientific Inc., Waltham, MA, USA) for 30 min at 25 °C. Subsequently, the samples were sonicated using an ultrasonic bath (Thermo Fisher Scientific Inc., Waltham, MA, USA) for 30 min at 25 °C. Finally, the samples were centrifugated in a Hermle centrifuge Z326K (Hermle Labortechnik GmbH, Wehingen, Germany) at 11,000 rpm for 15 min. Supernatants were collected. The extraction was repeated two times more on the solid residue. The three supernatants were mixed and stored until further analysis.

Aliquots of 50 µL of extract were added to 1950 µL of DPPH solution and were incubated for 30 min at room temperature. The absorbance was read at 515 nm (Jenway 6705UV/Vis, Cole-Parmer, Vernon Hills, IL, USA). A trolox calibration curve was prepared in the range of 150–750 mM, and the results were expressed as the µg Trolox equivalent per gram dry weight (dw).

#### 2.5.3. Total Phenolic Compounds

The method of Singleton and Rossi [45] and Campos et al. [19] was used to determine the total phenolic compounds with some modifications. In total, 100 μL of the extract was mixed with 900 μL of Folin–Ciocalteu reagent. The solution was allowed 5 min at room temperature, and then it was added to 700 μL of 7% sodium carbonate solution in distilled water. It was mixed and incubated for 90 min, and the absorbance was read at 725 nm. Gallic acid was used as a standard for the calibration curve. The total phenolic compounds were expressed as mg of gallic acid equivalent (GAE) per gram.

#### 2.5.4. Enzymatic Activity

The oxidation of the catechol by the polyphenol oxidase determines the enzymatic activity in a matrix such as avocado pulp. The enzymatic activity (*E.A.*) of the polyphenol oxidase was determined by using 0.2 mL of the sample, 2.4 mL of the buffer of phosphates (10 mM, pH 6.5), and 0.4 of catechol (0.5 M). The change in absorbance was determined spectrophotometrically (Jenway 6705UV/Vis, Cole-Parmer, Vernon Hills, IL, USA) at 420 nm for 10 min every 30 s and 20 °C [46].

The reaction rate for the polyphenol oxidase was defined by the change in the absorbance for *t* = 1 min (Equation (3)):

(3) $$E.A.=\frac{{∆A}_{420\;nm}}{t}$$

### 2.6. Microstructure Analysis

#### 2.6.1. Differential Scanning Calorimetry

The isothermal determinations were performed in a differential scanning calorimetry (DSC) equipment (Diamond DSC, Perkin-Elmer, Waltham, MA, USA). Hermetic aluminum pans were used to process the samples. The setting conditions for the analysis were as follows: holding for 1 min at −20 °C; heating the sample from −20 °C to 80 °C at a rate of 10 °C/min. The following configuration was intended to imitate the processing conditions of the heating and cooling stages: holding for 1 min at 20 °C, heating to 80 °C, and cooling up to −20 °C at a rate of 10 °C/min.

#### 2.6.2. X-Ray Diffraction

The samples of avocado pulp stored at three temperatures (4, 10, and −20 °C) were analyzed in a Rigaku MiniFlex (Rigaku Holdings Corporation, Tokyo, Japan) diffractometer to obtain the XRD patterns with Cu Kα radiation (λ = 1.540 56 Å). The results of the XRD analysis were the diffraction angles 2θ, intensity, and d-spacing.

#### 2.6.3. HRTEM

The avocado samples (approximately 10 µL) were placed on a carbon grid coated with formvar and analyzed in an Aberration Corrected Cold Field Emission Scanning Transmission Electron Microscope Jeol JEM-ARM200CF (JEOL Ltd., Tokyo, Japan). The microscope was equipped with a cold field emission gun, Cs corrector, and a high-angle annular dark field (HAADF) detector and had an ultra-high resolution of 0.72 Å. An electron beam spot with a condenser aperture of 60 nm at 200 kV for less than 30 s was used. Several locations on individual samples were analyzed. Fast Fourier Transform (FFT) analysis and image processing were applied using the freely available Digital Micrograph (GATAN Inc., Pleasanton, CA, USA) software (https://www.gatan.com/products/tem-analysis/gatan-microscopy-suite-software, accessed on 18 November 2024) attached at the microscope.

## 3. Results

### 3.1. Physicochemical Characterization of Avocado Pulp

Table 3 summarizes the physicochemical characterization of the avocado pulp before and after SSHE processing. In the case of the color difference, the first result shown is after comparing the raw pulp and the avocado pulp before the process. The ΔE* after processing and two weeks of storage at 4 °C was calculated with respect to the color of the raw pulp.

The average temperature of avocado pulp at the heating stage was approximately 80 °C. However, the residence time inside each SSHE was short (approximately 5 min) to minimize the effect of the high temperature on the bioactive compounds. Also, the temperature of heating can impact the evaporation of water and, in turn, impact the crystallization process. For example, in some products, such as with the crystallization of sugars, there is a desirable decrease in the aqueous content, given that drying influences the rate of crystallization of sugars [32]. In the case of avocado pulp, the moisture content is relevant to the re-emulsification process in the pre-cooling and cooling stages.

**Figure 2:**
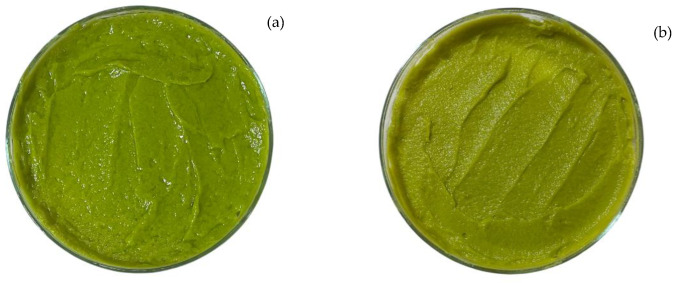
Avocado pulp (**a**), and avocado pulp processed and stored at 4 °C for 2 weeks (**b**).

The pigments in avocado pulp are chlorophylls and carotenoids [47,48]. The temperature, pH, and mechanical forces can degrade these compounds and change their color during storage or after processing [13]. The first compounds of the chlorophyll breakdown are pheophytins and pheophorbides [49,50]. As a result, the avocado pulp showed ‘degreening’ [50]. To the naked eye, the color change seemed non-drastic. The crystallization process could have delayed the degradation kinetics of the pigments, and the final product was more stable than the avocado pulp. Figure 2 shows the avocado pulp after process (a), and the avocado pulp processed and stored at 4 °C (b). The product stored at 10 °C remained without signs of discoloration or spoiling for 2.5 weeks. Discoloration and spoilage was not evident in the samples stored at 4 °C and −20 °C after 5 weeks. 

#### Viscosity

The viscosity of the avocado pulp, according to the power law model, indicated a pseudoplastic behavior, given the behavior index (n) was 0.349 ± 0.05 and the consistency index was 0.854 ± 0.04 Pa·s. In crystallization, high viscosities could make it difficult to reach a thermodynamic steady state in the sample. The viscosity of the avocado pulp could affect the nucleus formation and crystal growth. For instance, in products such as caramels with high sucrose or lactose, and candies, the high viscosity inhibits the rate of nucleation [32]. However, Sonwai et al. [51] correlated the increase in viscosity with the degree of crystallinity in the product, and with increased steady shear the crystallization was faster and the degree of crystallinity was higher. Viscosity might be an important parameter influencing the crystallization degree. Furthermore, the measurement of viscosity during storage could be considered as a control parameter for crystallization.

### 3.2. Biochemical Characterization

#### 3.2.1. Oil Content

The fat content in the avocado puree immediately after SSHE processing was 24.3 ± 0.9%. Variations in the dry matter and fat content of avocado pulp are due to geographical location, growing conditions, maturity stages, environmental conditions, and genetics [19,52,53]. These results were relevant due to the possible generation of oil-in-water emulsions by effect of the thermal and mechanical stress involved in SSHE processing, as reported in different food products [38,54] and which could be important for the crystallization observed in the avocado pulp.

#### 3.2.2. Antioxidant Capacity and Total Phenolic Compounds

The antioxidant capacity determined by the DPPH method before and immediately after the processing through the SSHE system was 0.135 ± 0.002 ^a^ µg TE/g dw and 0.108 ± 0.001 ^b^ µg TE/g dw, respectively. The total phenolic content was 6.024 ± 0.213 ^c^ mg GAE/g dw of avocado pulp and 4.571 ± 0.263 ^d^ mg GAE/g dw after processing. The decrease in the antioxidant capacity was probably due to the thermal treatment with the SSHE system. However, the DPPH method can be affected by how antioxidants act in the electron transfer due to the exposure of light or oxygen and the interaction of water [55].

The variations of the total phenolic compounds and the antioxidant capacity reported for the avocado pulp fluctuate with different stages of fruit maturity, the geographical location of the fruit, and the process employed for the avocado pulp [56], such as the use of a high-pressure [47], lyophilization [25], cold plasma [55], and pulse light [57]. The results of the increase or decrease in the antioxidant capacity by effect of these different technologies are related to the operating conditions and the residence time associated with the process [58].

#### 3.2.3. Enzymatic Inactivation

**Figure 3:**
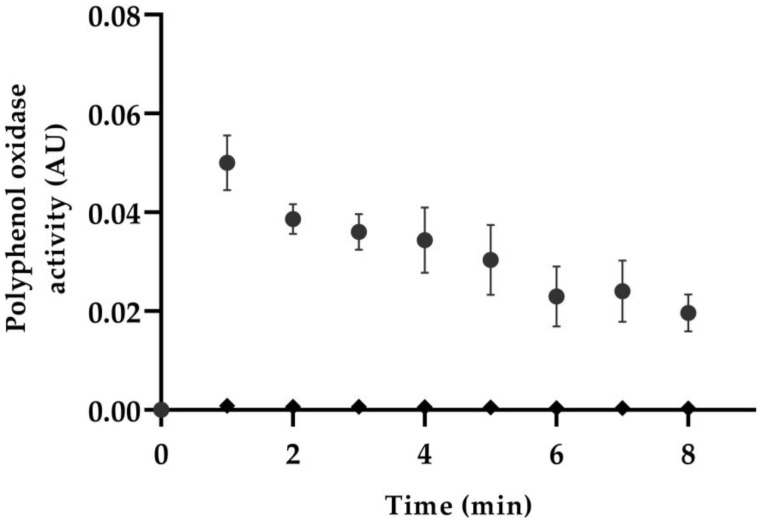
Enzymatic activity of polyphenol oxidase of avocado pulp and avocado pulp after SSHE processing. Circles represent the enzymatic activity found in the avocado pulp before the process, whereas rhombi are for the avocado pulp after processing.

The change in the absorbance found in the avocado pulp sample shows the polyphenol oxidase reaction to the catechol, which causes enzymatic browning in the avocado (Figure 3). However, the thermal treatment in the SSHE inactivates the enzymatic activity because there are no changes in the absorbance of the avocado pulp processed.

The activity of the enzyme polyphenol oxidase (PPO) is undesirable in some vegetables and fruits, such as avocado pulp. Generally, the browning formation on the avocado pulp is associated with a loss in the nutritional value of the fruit, mainly in the proteins, amino acids, or lipids, due to the formation of free radicals during the reduction of ο-quinone molecules in the redox reaction [26,59]. The damage of the pulp’s color and the decrease in the bioavailability of the nutrients is an indicator of poor quality in avocado pulp products [26,60,61]. The efforts of the food industry to extend the shelf-life of the avocado mesocarp focused on reducing or inactivating the activity of PPO.

### 3.3. Microstructure

#### 3.3.1. Differential Scanning Calorimetry

**Figure 4:**
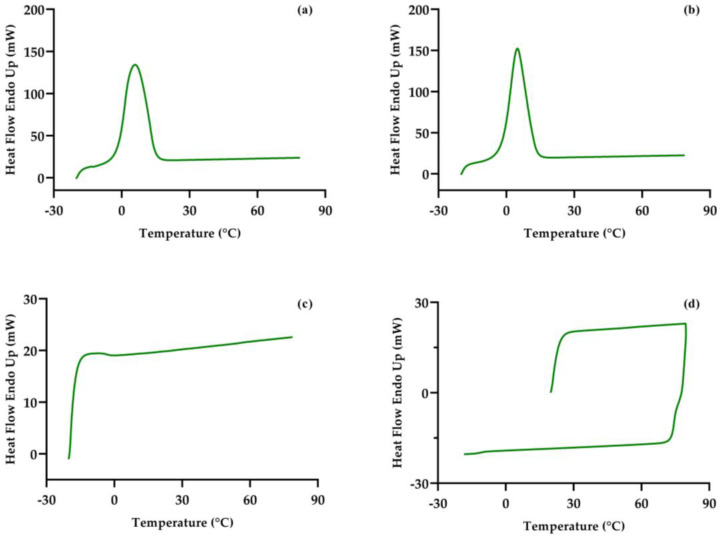
DSC profiles of avocado pulp immediately after processing (AP) (**a**), avocado processed and stored at 4 °C (AP_P4_) (**b**), avocado processed and stored at −20 °C (AP_P-20_) (**c**), and avocado without processing and heated and cooled for comparison (**d**).

The temperatures of the processing were imitated in the DSC analysis in order to analyze whether there was a phase transition in the avocado pulp with the thermal shock. Nonetheless, the thermograms obtained do not correspond to the real processing conditions because during the DSC analysis it is not possible to include the effect of the convective heat transfer (see Figure 4). The mechanical action of the blades in similar systems have been reported to separate the oil; thus, the temperature differences as well as the blade rotation involved in the process seemed to produce the reincorporation of the oil drops into the aqueous matrix of the avocado pulp, i.e., re-emulsification. Also, the composition of avocado pulp and the variety of fatty acids present in avocado oil does not allow for the obtaining of defined peaks that would indicate the phase transition. Table 4 shows the peaks for avocado without processing and with storage at 4 °C, and the temperatures for every sample are similar; however, in the case of the sample stored at −20 °C, a change in the behavior was observed; specifically, the DSC showed a possible second-order transition of the avocado pulp after storage at −20 °C.

Tan et al. [2] obtained the same peaks for avocado oil and suggested the correlation with the degree of saturation and the temperature of the melting peaks of tri-saturated and mono-unsaturated TAGs, compared with the tri-unsaturated TAGs, which have a lower melting point and possibly generate unstable crystals.

#### 3.3.2. X-Ray Diffraction and HRTEM

**Figure 5:**
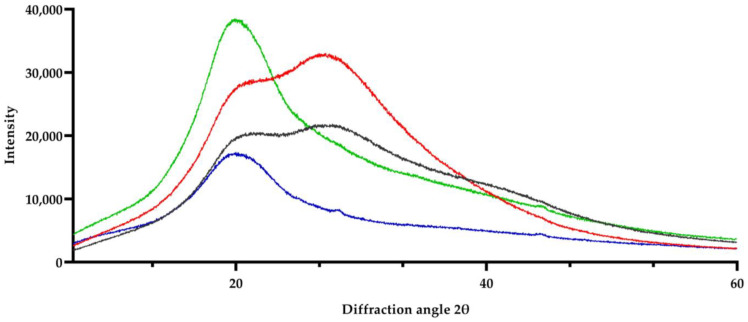
XRD patterns: green plot: avocado pulp without process; blue plot: avocado processed and stored at 10 °C; red plot: avocado processed and stored at 4 °C; and gray plot: avocado processed and stored at −20 °C.

Figure 5 displays the diffraction patterns of the samples obtained from the three different conditions of avocado pulp processing under the prepared SSHE system. With respect to the nuclei formation process, the nuclei can be affected by surfactants (such as saturated or unsaturated mono- and diacylglycerols) and by the affinity to the hydrophobic tail between the surfactant molecule structure and the triacyl glycerols (TAGs) [62,63,64,65].

The oleic and palmitic acids present higher concentrations in avocado Hass (*Persea americana* Mill.), followed by linoleic and palmitoleic acids [36,41,66,67,68]. The TAG structure, the carbon number in TAGs and diversity of fatty acids, as well as the presence of specific TAGs would affect the arrangement of the polymorphic form [63,69,70,71]. The operating conditions are also relevant for the formation of nuclei during the process, as well as the storage conditions [32]. Thus, the diffraction patterns show the differences in the intensity of the polymorphs for the three avocado samples processed and stored at different temperatures. According to the measurements for each peak, in the case of the avocado pulp processed and stored at 4 °C, the interplanar spacings with the highest intensity were at *d* = 4.217 Å.

As compared to the reports of interplanar spacings for fatty acids, the polymorph type with the higher proportion in avocado pulp processed and stored at 4 °C was β′. Water and triglycerides are the major components of avocado pulp [72]. Therefore, these components are the most involved in the formation of nuclei. In agreement with the triacyl glycerol profile of avocado, storage at 4 °C favored the growth of β′ polymorphs. In products such as margarine or cocoa butter, the form β′ is desirable because it is associated with a smooth and creamy texture [71]. In the case of cocoa butter, monosaturated fatty acids are predominant (palmitate, oleate, and stearate) and promote six different types of the polymorph β′ [51].

The effect of rotor speed and temperature contribute to the rupture of idioblastic cells of the avocado, and to the release of the oil contained in the cells into the avocado matrix. As explained above, in the pre-cooling phase of the process, the lower temperature contributed to the re-emulsification of the avocado oil in the pulp matrix. Under the cooling temperature, the nucleation of triglycerides and other components of the avocado pulp, such as fiber, carbohydrates, water, and proteins, was triggered.

Table 5 shows the distribution of different types of polymorphs across the diffractogram of avocado samples. The form β′ is the polymorph with the highest intensity, followed by the form α. Due to the rotation speed, storage temperature, and the fatty acid profile, the polymorph α present in the avocado pulp would change into the form β after a longer time of crystal maturation.

**Figure 6:**
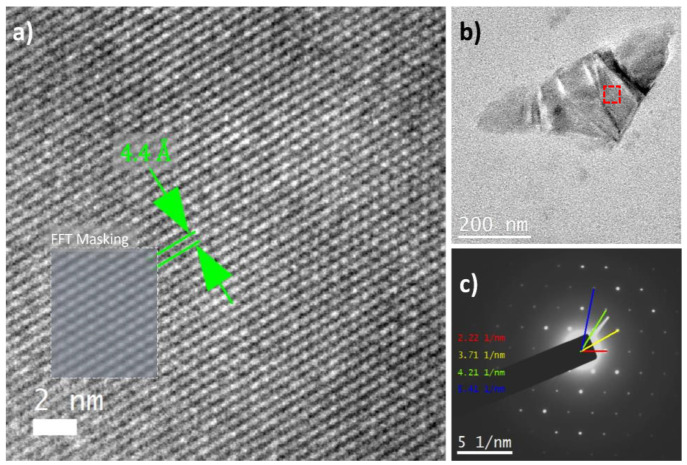
TEM micrographs (**a**,**b**) and SAED pattern of avocado pulp processed and stored at 4 °C. (**a**) Region enclosed by the red box in the micrograph (**b**). (**c**). Fast Fourier Transform (FFT) masking tool shows atomic columns as found in the region indicated by the gray square.

Figure 6 shows the selected-area electron diffraction (SAED) pattern for the avocado pulp processed and stored at 4 °C. The presence of the geometry’s bright spots (Figure 6c) indicates the formation of crystals in the avocado pulp. The change in the microstructure of the avocado pulp and its consequences on its shelf-life have not been reported yet. The composition of the avocado fruit, operating conditions, and temperature storage contributed to the formation of nuclei and maturation of the different types of crystals [63,73].

**Figure 7:**
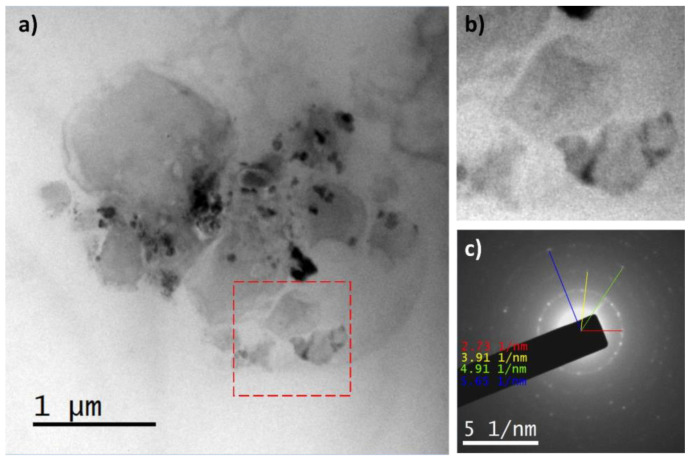
TEM micrographs (**a**,**b**) and SAED pattern of avocado pulp processed and stored at 10 °C (**c**). (**b**) Region enclosed by the red box in the micrograph (**a**).

In the case of the avocado pulp processed and stored at 10 °C, the highest intensity was for polymorph β, as shown in Table 6. However, the presence of polymorphs α and β, for an equal intensity, gave an SAED pattern that is shown in Figure 7.

The stability of the crystals and the definition in the SAED pattern were not equal to that of the avocado pulp stored at 4 °C, because the temperature of maturation allows the growth of crystals but not with enough intensity to make the pulp stable. Also, the decomposition of the avocado pulp was faster than that of the avocado pulp stored at 4 °C.

**Figure 8:**
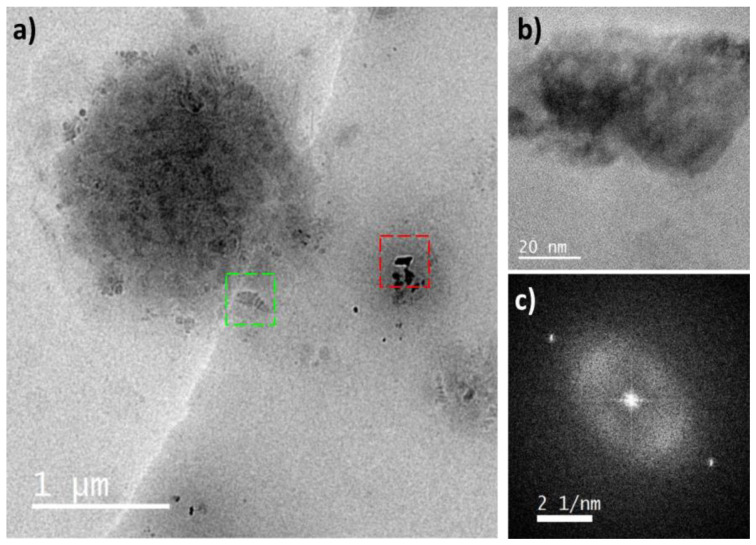
TEM micrographs (**a**,**b**) and FFT pattern of avocado pulp processed and stored at −20 °C in the region enclosed by the red box (**c**). (**b**) Region enclosed by the red box in the micrograph (**a**). The region enclosed by the green box shows particles with similar morphology to those found in samples stored at −4 °C and 10 °C.

The FFT pattern of the avocado pulp processed and stored at −20 °C (see Figure 8) showed only two bright spots, and if it is compared with the results in Table 7, the intensity of alpha and beta forms was equal; hence, the stability of the crystals did not allow their distinction by TEM. The storage temperature affected the growth of polymorphs, and due to the composition of the avocado pulp, water was probably frozen and did not allow the maturation of the polymorphic forms.

Several studies have reported that α crystals are composed of long, saturated fatty acid molecules. Given the cell breakage induced by the SSHE processing, which leads to the release of oil from idioblastic cells, the formation of these polymorphs could be triggered briefly after the first stage of the SSHE processing; that is, where temperature is still high and such kinds of fatty acid chains would have a greater probability to intercalate and form the crystal networks, which could also be structures with a lower density and prone to be reorganized into more stable crystals. In the second and third stages of the SSHE process applied, a consequence of the lower temperature and emulsification of the oil released from the idioblastic cells could be the tighter packing of the more stable polymorph β′. Therefore, the formation of the β polymorphs could be the result of the crystal maturation occurring under storage conditions, i.e., once the pulp was processed by the SSHE. Nevertheless, an optimal temperature would be required for the formation of the more stable polymorphs, given that their presence was markedly increased when the storage temperature was 4 °C rather than 10 °C or −20 °C [74,75].

## 4. Conclusions

Polymorphs in avocado pulp were identified after the implementation of scraped-surface heat exchanger processing. The type of polymorphic growth in avocado pulp with the highest intensity was β′. This process allowed the formation of polymorphs in the avocado pulp, particularly when the pulp was stored at 4 °C. Blade rotation speed affects the development of nuclei, and the storage temperature had an impact on the maturation of different polymorphic forms.

This process represents a new alternative to extend the shelf-life and change the microstructure of avocado puree. The polymorphism in the avocado puree preserved the color and improved the sensorial characteristics of the final product through a smooth and creamy texture. It is important to explore the relationship between the polymorphism found through the process studied here and the inactivation of the polyphenol oxidase enzyme, to minimize the losses of the bioactive compounds.

## Figures and Tables

**Table 1:** Interplanar spacing values reported for fatty acids [27,28,31].

Polymorphic Form	Interplanar Spacing (Å)	2θ Angle	Melting Points	Packing
α	4.15	Long spacings: 1–15° Short spacings: 16–25°	Lower	Hexagonal
β′	4.2–4.3 and 3.7–4.0		Intermediate	Orthorhombic
β	4.6		Higher	Triclinic

**Table 2:** Operating conditions of the three different stages of the process.

	Stage		
Condition	Heating	Pre-Cooling	Cooling
Medium	Thermal oil	Water	Water
Mean temperature (°C) of the medium	150	8	4
Residence time (min)	4	4	5
Blade rotation speed (rpm)	300	200	200

**Table 3:** Results of physicochemical parameter of avocado pulp.

Parameter	Before SSHE Processing	After SSHE Processing
Moisture content (%)	61 ± 2.8 ^a^	57 ± 1.3 ^a^
Color difference, ΔE* (dimensionless)	1.89 ± 0.07 ^b^	5.02 ± 0.06 ^c^

Results are mean SD (*n* = 3). Means within row with different superscript small letters are statistically different (*p* < 0.05).

**Table 4:** The DSC results of the avocado pulp and avocado pulp processed and stored at 4 °C.

Sample	T_OS_ (°C)	T_E_ (°C)	Peak (°C)	ΔH (J/g)
A_Pulp_	−1.75	14.90	5.92	252.2326
A_4_	−1.75	12.86	4.87	238.3095

**Table 5:** Interplanar spacing values for avocado pulp processed and stored at 4 °C.

Polymorphic Form	Interplanar Spacing (Å)	2θ Angle	Intensity Counts
α	4.15	21.353	28,410
β′	4.329 4.055 3.895 3.701	20.502 21.900 22.812 24.027	28,341 28,685 28,960 29,648
β	4.686	18.921	25,108

**Table 6:** Interplanar spacing values for avocado pulp processed and stored at 10 °C.

Polymorphic Form	Interplanar Spacing (Å)	2θ Angle	Intensity Counts
α	4.158	21.353	16,303
β′	4.316 4.229 4.089 3.701	20.562 20.988 21.717 24.027	17,059 16,647 15,477 11,212
β	4.613	19.225	17,059

**Table 7:** Interplanar spacing values for avocado pulp processed and stored at −20 °C.

Polymorphic Form	Interplanar Spacing (Å)	2θ Angle	Intensity Counts
α	4.158	21.353	20,430
β′	4.217 3.835 3.701	21.049 23.835 24.027	20,361 20,499 20,361
β	4.613	19.225	18,366

## Data Availability

The original contributions presented in the study are included in the article, further inquiries can be directed to the corresponding author.
